# Supplementary material for: Hepatic stellate cells control liver zonation, size and functions via R-spondin 3
Source: Nature. 2025 Mar 12;640(8059):752–61. doi: 10.1038/s41586-025-08677-w (PMC12003176; doi:10.1038/s41586-025-08677-w)
Supplement: Supplementary file 1 — Supplementary Figs. 1–6. [file 41586_2025_8677_MOESM1_ESM.pdf]

nature portfolio

https://doi.org/10.1038/s41586-025-08677-w

# Supplementary information

# Hepatic stellate cells control liver zonation, size and functions via R-spondin 3

In the format provided by the authors and unedited

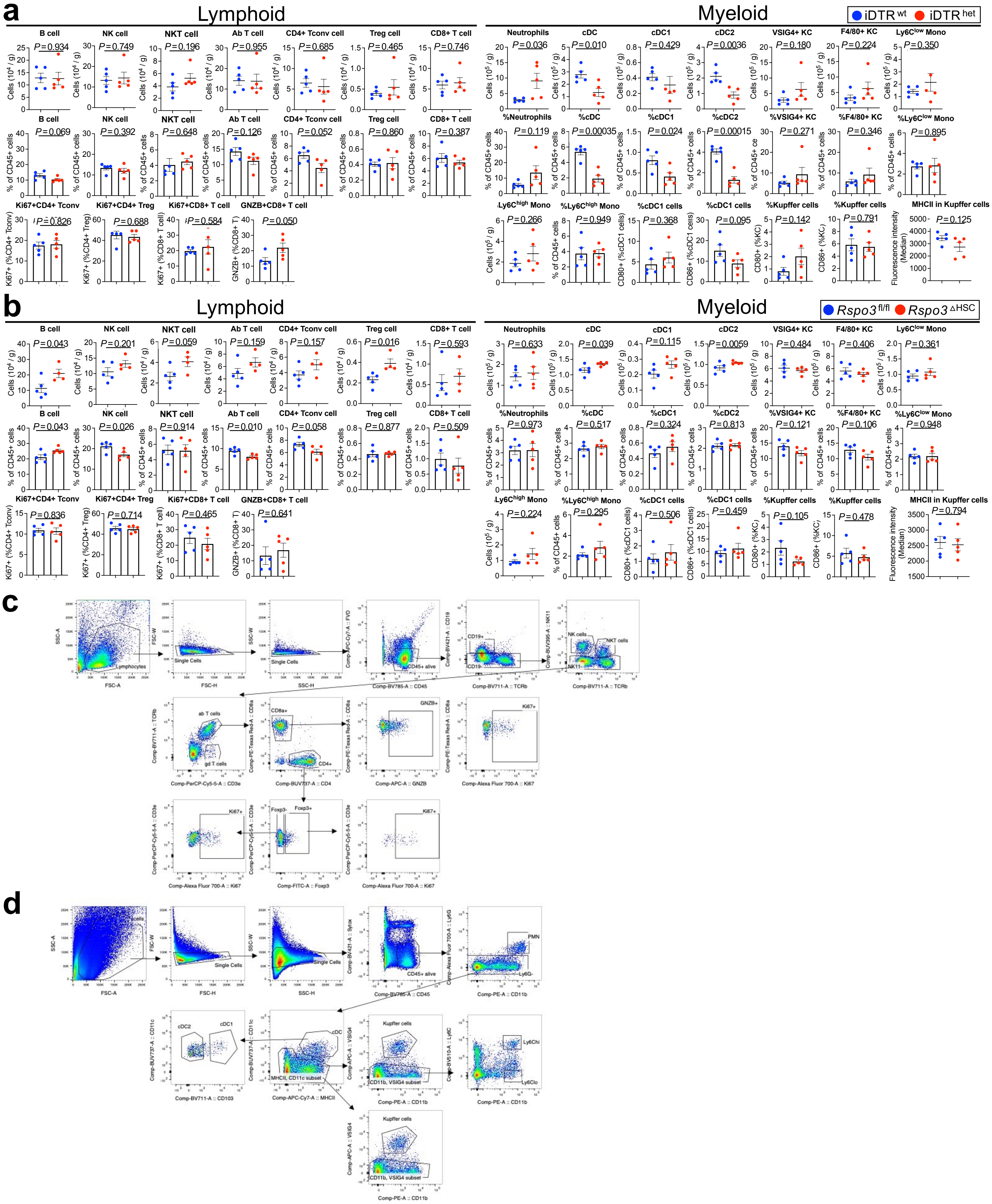


**Supplementary Information 1| Immune cell analysis by flow cytometry in normal mouse livers from mice with or without genetic HSC depletion and in normal livers from mice with genetic deletion of Rspo3 in HSCs. a.** Seven days after DT injection, iDTR$^{wt}$ and iDTR$^{het}$ mice (*n*=5/group) were euthanized. Lymphoid and myeloid cell populations were isolated from the liver and quantified by FACS analysis. **b.** FACS analysis of immune cells in *Rspo3*$^{fl/fl}$ (*n*=5) and *Rspo3*$^{\Delta HSC}$ mice (for cell number *n*=4; for cell% *n*=5). **c-d.** Representative images of the gating strategy used to analyse lymphocyte (**c**) and myeloid cell (**d**) populations. Data are shown as mean ± s.e.m. Each dot represents one biological replicate (**a**,**b**). P-values were calculated using unpaired two-tailed t-tests (**a**,**b**). NK: Natural Killer, NKT: NK T cells, Tconv: Conventional T, Treg: Regulatory T, DC: Dendritic cell, KC: Kupffer cells and Mono: Monocytes.

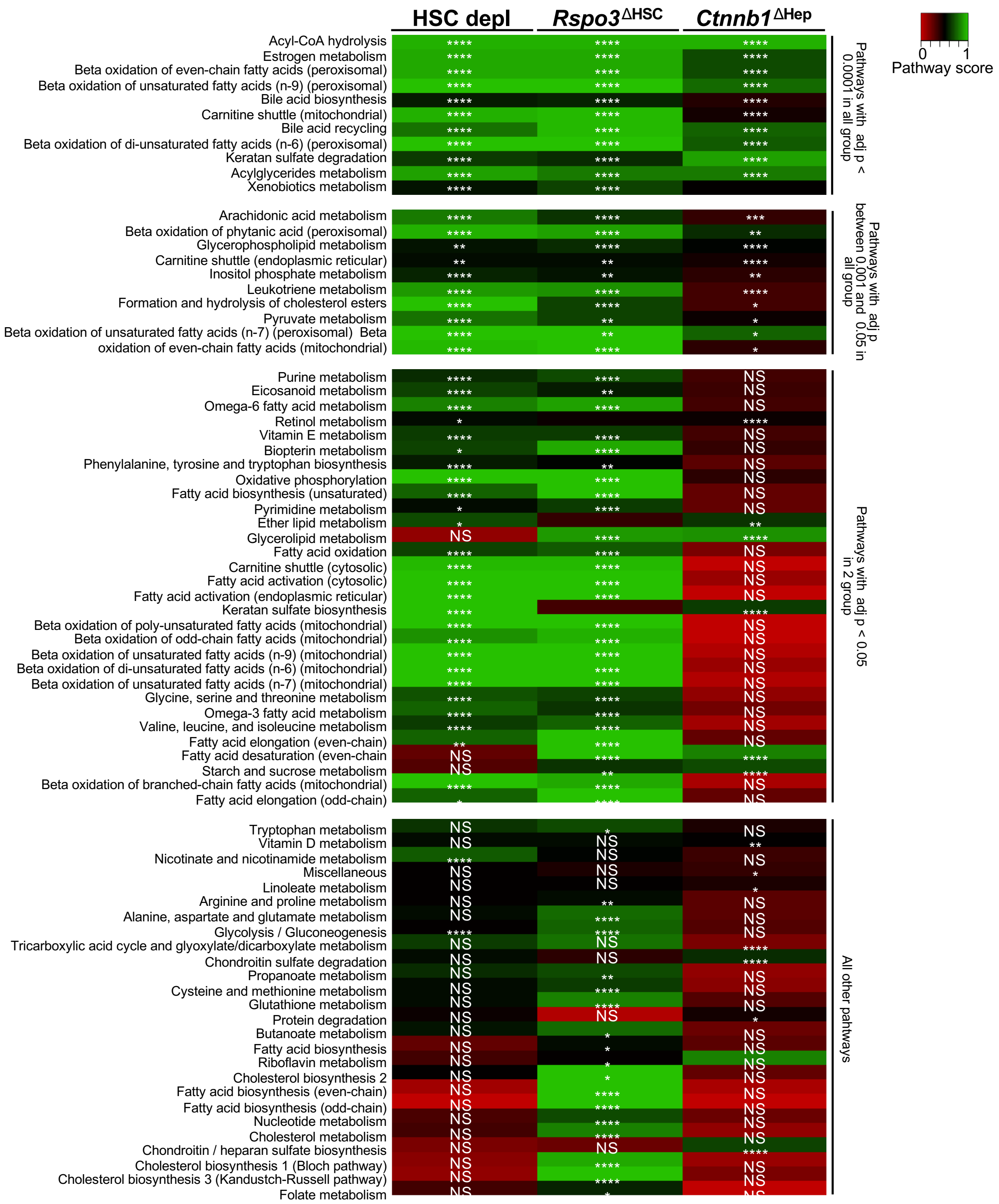


**Supplementary Information 2| In silico metabolomic analysis in transcriptomic data from HSC-depleted liver, hepatocytes isolated from *Rspo3*$^{\Delta HSC}$ livers, and *Ctnnb1*$^{\Delta Hep}$ livers.** Transcriptomics driven metabolic pathway analysis (TDMPA) was done using RNA-seq data comparing HSC-depleted (JEDI) to control livers, snRNA-seq comparing hepatocytes from *Rspo3*$^{\Delta HSC}$ and *Rspo3*$^{fl/fl}$ mice and microarray data, comparing *Ctnnb1*$^{\Delta hep}$ to *Ctnnb1*$^{fl/fl}$ livers. Analysis is displayed as heatmap of 77 pathways in the three groups. *P < 0.05; **P < 0.01; ***P < 0.001; ****P < 0.0001. NS, non-significant. P-values were calculated using the hypergeometric test with false discovery rate correction according to Benjamini and Hochberg.

**a**

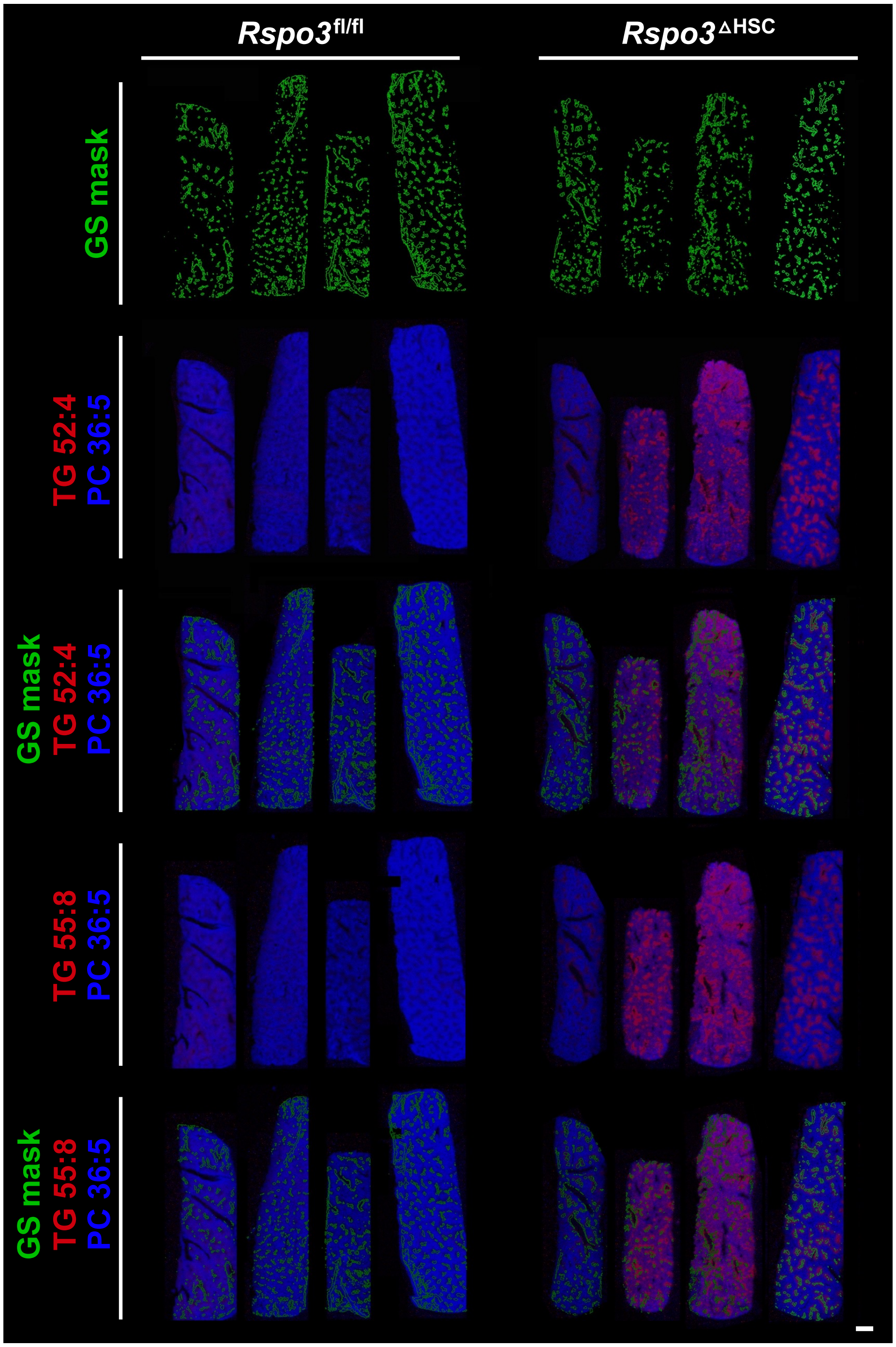


**b**

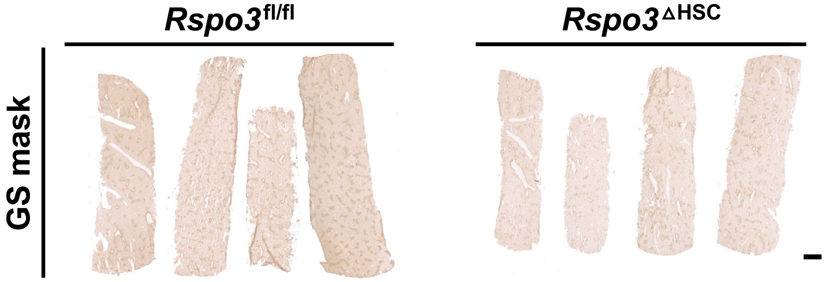


**Supplementary Information 3| Desorption ionization mass spectrometry imaging of triglycerides in *Rspo3*$^{\Delta HSC}$ and *Rspo3*$^{fl/fl}$ livers. a.** DESI imaging in *Rspo3*$^{\Delta HSC}$ and *Rspo3*$^{fl/fl}$ livers (*n*=4 each) for 52:4 and 55:8 triglyceride species, also showing PC36:5 as well as an overlay with glutamine synthetase (GS) as a marker of pericentral location. **b.** Immunohistochemistry for GS used for determination of the GS mask in (**a**). Scale bar 1 mm (**a**,**b**).

**a**

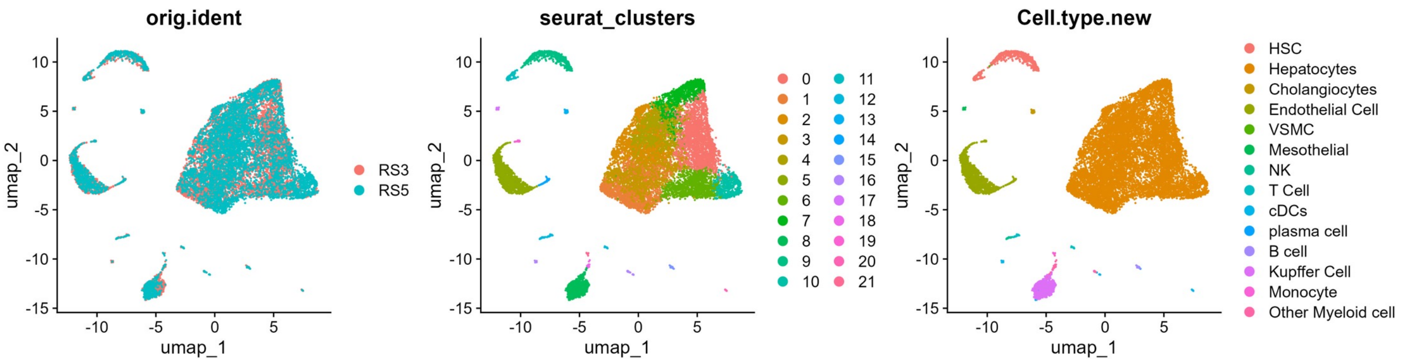


**b**

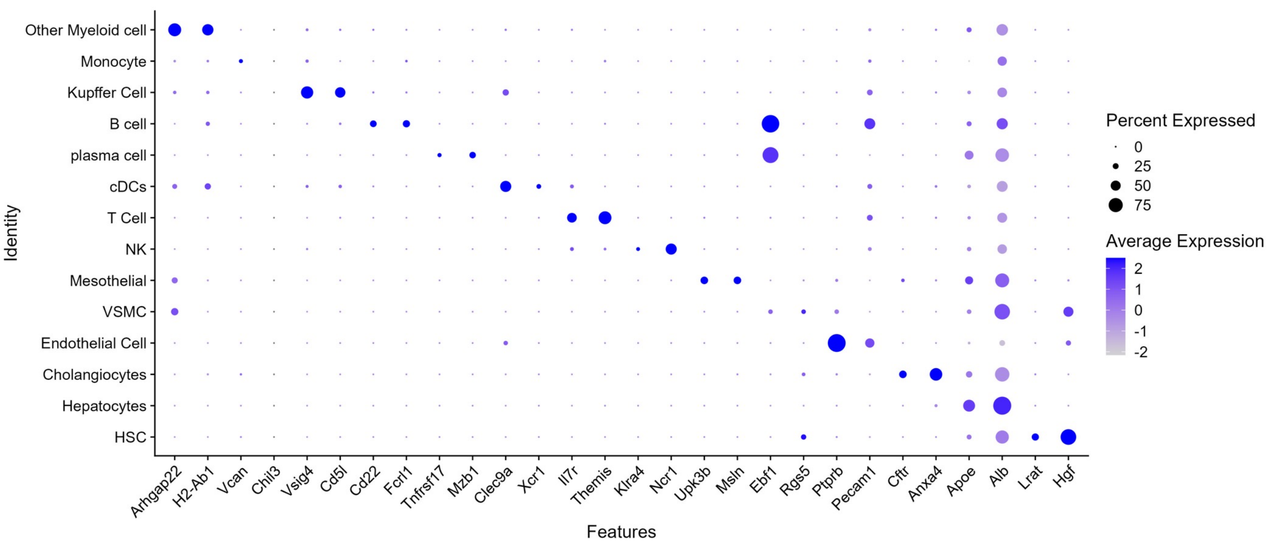


**Supplementary Information 4| Single nucleus RNA-sequencing analysis of mouse liver tissue. a.** UMAP visualization of sn-RNAseq of mouse livers (RS3 = Cre-negative iDTR$^{wt}$, RS5 = Cre-negative Rspo3 $^{fl/fl}$). Clusters (middle) were subset and analysed to determine cell populations (right) using two marker genes for each cell type. **b.** Dotplot showing two marker genes for each cell population.

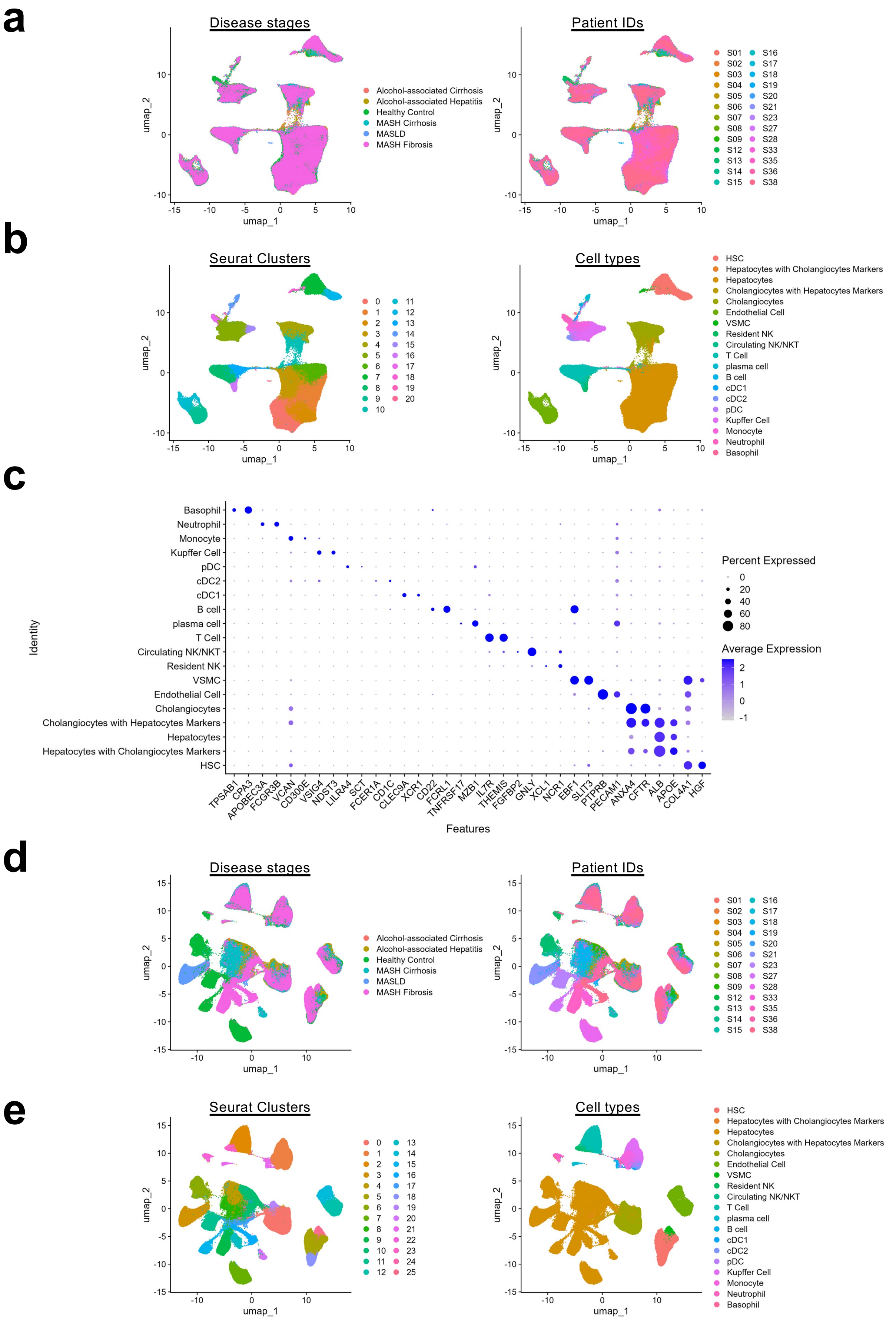


**Supplementary Information 5| Single nucleus RNA-sequencing analysis of human liver tissue. a.** Batch-corrected UMAP of sn-RNAseq of human livers, visualizing disease stage [left panel, normal (n=6), alcoholic cirrhosis (n=4), alcoholic hepatitis (n=5), MASLD (n=3), MASH fibrosis (n=4) and MASH cirrhosis (n=4) cases] and patient ID (upper right panel). **b.** Clusters (left panel) were subset and analysed to determine cell populations (right panel). **c.** Dotplot shows 2 specific marker gene for each cell type. **d-e.** Not batch-corrected UMAP visualization of the same dataset showing disease stage and patient ID (**d**) as well as Seurat clusters, and cell types (**e**).

**a**

## *Full size immunoblots Extended Data Figure 6i*

**Western blot for Rspo3 (9/19/2024)**

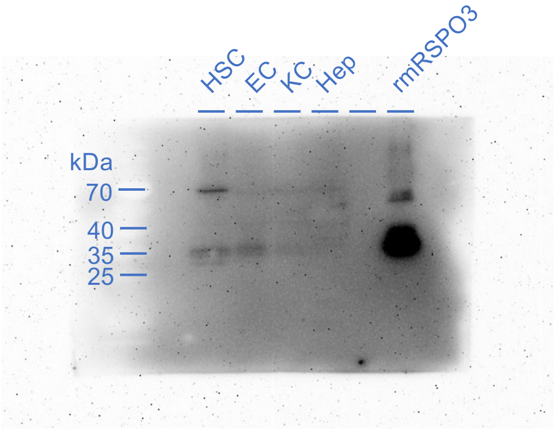


**Western blot for beta-actin (9/19/2024)**

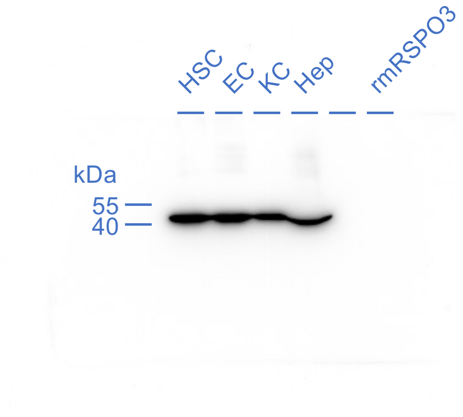


**b**

## *Full size immunoblots Extended Data Figure 10f*

**Western blot for Aldh2 (12/27/2023)**

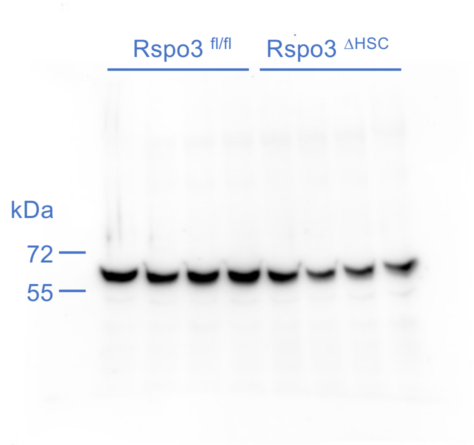


**Western blot for GAPDH (12/27/2023)**

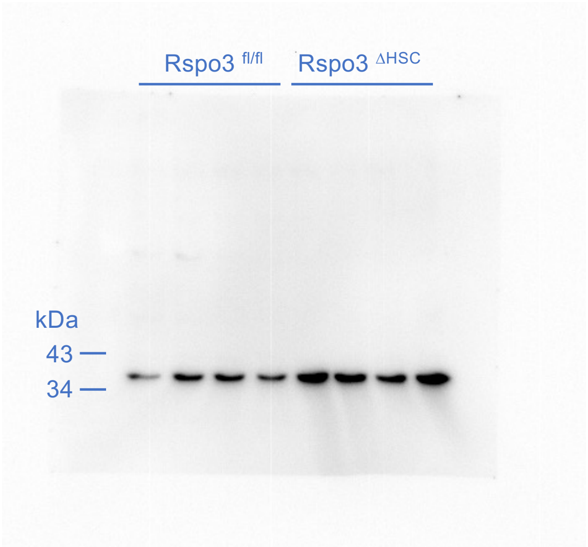


**Supplementary Information 6| Uncropped immunoblots from Extended Data Figure 6i and Extended Data Figure 10f. a-b.** Uncropped immunoblots showing Rspo3 (left panel) and beta-actin (right panel) from Extended Data Figure 6i (**a**) and immunoblots showing Aldh2 (left panel) and GADPH (right panel) from Extended Data Figure 10f (**b**). HSC: Hepatic stellate cells, EC: Endothelial cells, KC: Kupffer cells, Hep: Hepatocytes and rmRSPO3: Recombinant mouse RSPO3 protein.
