# Supplementary material for: Hepatic stellate cells control liver zonation, size and functions via R-spondin 3
Source: Nature. 2025 Mar 12;640(8059):752–61. doi: 10.1038/s41586-025-08677-w (PMC12003176; doi:10.1038/s41586-025-08677-w)
Supplement: Supplementary file 2 — Reporting Summary [file 41586_2025_8677_MOESM2_ESM.pdf]

Corresponding author(s): Robert Schwabe

Last updated by author(s): Nov 26, 2024

Reporting Summary

Nature Portfolio wishes to improve the reproducibility of the work that we publish. This form provides structure for consistency and transparency in reporting. For further information on Nature Portfolio policies, see our Editorial Policies and the Editorial Policy Checklist.

Statistics

For all statistical analyses, confirm that the following items are present in the figure legend, table legend, main text, or Methods section.

n/a	Confirmed	
☐	☒	The exact sample size (*n*) for each experimental group/condition, given as a discrete number and unit of measurement
☐	☒	A statement on whether measurements were taken from distinct samples or whether the same sample was measured repeatedly
☐	☒	The statistical test(s) used AND whether they are one- or two-sided<br>*Only common tests should be described solely by name; describe more complex techniques in the Methods section.*
☒	☐	A description of all covariates tested
☐	☒	A description of any assumptions or corrections, such as tests of normality and adjustment for multiple comparisons
☐	☒	A full description of the statistical parameters including central tendency (e.g. means) or other basic estimates (e.g. regression coefficient) AND variation (e.g. standard deviation) or associated estimates of uncertainty (e.g. confidence intervals)
☐	☒	For null hypothesis testing, the test statistic (e.g. *F*, *t*, *r*) with confidence intervals, effect sizes, degrees of freedom and *P* value noted<br>*Give P values as exact values whenever suitable.*
☒	☐	For Bayesian analysis, information on the choice of priors and Markov chain Monte Carlo settings
☒	☐	For hierarchical and complex designs, identification of the appropriate level for tests and full reporting of outcomes
☐	☒	Estimates of effect sizes (e.g. Cohen's *d*, Pearson's *r*), indicating how they were calculated

Our web collection on statistics for biologists contains articles on many of the points above.

Software and code

Policy information about availability of computer code

Data collection	Bulk and single cell RNA-sequencing data was generated from frozen mouse or frozen human livers as described in the method section.<br>The following instruments were used for data collection: NovaSeq 6000 (Illumina); FluorChem M system instrument (ProteinSimple); QuantStudioTM5 Real-Time PCR System (Applied Biosystems); NanoDropTM 1000 Spectrophotometer (Thermo Scientific); Hybridization System (ACDbio); Bioanalyzer 2100 (Agilent Technologies); iMarkTM Microplate Reader (BioRad).
Data analysis	Details for data analysis are described in the Material and Methods. The following softwares/packages were used for data analysis: Kallisto (0.44.0); Cell Ranger (v3.1.0); CellBender (v.0.2.0); R (v4.0.2); RStudio (2023.12.0 build 369); R package survminer (0.4.9); R package maxstat (v.0.7-25); R package DESeq2 (v 1.40.2); R package Seurat (v5.0.1); R package ggplot2 (v3.4.4); R package dplyr (1.1.4); WebIDQ software; Limma; GraphPad Prism (v.9.0); FIJI (v2.14.0); ilastik (v1.3.3post3); CellProfiler (v4.2.1); GSEA (v4.3.2); ggplot2 (v 3.4.4);HDI imaging software (v1.6); SCiLS (v2024a);Scrublet; CellphoneDB (v5)<br>The previously published datasets GSE68779, GSE211370, GSE172492, GSE158183, GSE49541, GSE193066, GSE192959, GSE103580 and GSE94397 were used for analysis. R markdown scripts enabling the main steps of the analysis have been deposited into GitHub (https://github.com/SchwabeLabcu/HSC_RSPO3).

For manuscripts utilizing custom algorithms or software that are central to the research but not yet described in published literature, software must be made available to editors and reviewers. We strongly encourage code deposition in a community repository (e.g. GitHub). See the Nature Portfolio guidelines for submitting code & software for further information.

Data

Policy information about availability of data

All manuscripts must include a data availability statement. This statement should provide the following information, where applicable:

- Accession codes, unique identifiers, or web links for publicly available datasets
- A description of any restrictions on data availability
- For clinical datasets or third party data, please ensure that the statement adheres to our policy

The snRNA-seq data from 26 human patients and 4 untreated mouse livers with the indicated genotypes (GSE256398); as well as bulk RNA-seq data from HSC-depleted vs control mice, Rspo3ΔHSC mice vs control mice, aged Rspo3ΔHSC mice vs control mice, and Rspo3ΔEC vs control mice (GSE256377) have been deposited.

Research involving human participants, their data, or biological material

Policy information about studies with human participants or human data. See also policy information about sex, gender (identity/presentation), and sexual orientation and race, ethnicity and racism.

Reporting on sex and gender	Sex and gender were not considered in the study design. The respective sex and gender information are detailed in the methods and figure legend and Supplementary Table 10
Reporting on race, ethnicity, or other socially relevant groupings	No further information about race, ethnicity, or other socially relevant groupings are reported.
Population characteristics	Human characteristics are reported in Supplementary Table 10
Recruitment	The respective recruitment process is listed in the Methods section.<br>All patients provided written informed consent. Liver samples for alcoholic hepatitis, alcohol-associated cirrhosis and MASLD-associated cirrhosis were obtained from liver explants in patients undergoing liver transplant. All other liver samples from living donors were obtained intraoperatively in patients in whom an intraoperative liver biopsy was indicated on clinical grounds such as during scheduled liver resection, exclusion of liver malignancy during major oncologic surgery, or assessment of liver histology during bariatric surgery.
Ethics oversight	The respective ethics committee is listed in the Methods section.<br>Human Liver samples were collected under IRB-approved protocols at the University of Pittsburgh (IRB protocol 19120198), Johns Hopkins University School of Medicine (IRB protocol 00107893), the University of Kiel, Germany (Ethikkommission der Medizinischen Fakultät der Universität Kiel, D425/07, A111/99) or obtained from LifeNet Health, an organ procurement organization (operating under the Anatomical Gift Act)

Note that full information on the approval of the study protocol must also be provided in the manuscript.

Field-specific reporting

Please select the one below that is the best fit for your research. If you are not sure, read the appropriate sections before making your selection.

☒ Life sciences ☐ Behavioural & social sciences ☐ Ecological, evolutionary & environmental sciences

For a reference copy of the document with all sections, see nature.com/documents/nr-reporting-summary-flat.pdf

Life sciences study design

All studies must disclose on these points even when the disclosure is negative.

Sample size	Pilot experiments and previously published results were used to estimate the sample size such that appropreate statistical tests could yield significant results. For some experiments, results were confirmed using a second cohort. The sample size and exact n numbers used in the study are indicated in the Methods or in each individual figure/figure legend.
Data exclusions	No mice were excluded because they were statistical outlier. However, some mice were excluded of the analysis if they presented sickness not related to the study (abscess, malocclusion of their teeth, infections due to fight, extensive hydronephrosis). Some samples were excluded if they did not meet quality standards, e.g. for qPCR when the house keeping gene 18s was more than 1 cycle lower than the mean, or for IHC when the staining was extremely faint or had high background, e.g. due to bad sample preparation or fixation. Some mouse samples were not analyzed if not enough tissues was collected.
Replication	All experiments presented were conducted with sufficient mouse numbers to ensure statistical significance could be reached, particularly for experiments involving tumor studies. Biochemical or image based data were reproduced in multiple mice; e.g. immunostainings, qPCR analysis, measurements of transaminase levels, immunoblotting experiments. All atempts of replicating data were successful. Number of mice, biological replicate and number of experiment represented in the figures are indicated in the figure legends.

Randomization	Individual were allocated in different experimental groups based on the expression of the Cre recombinase. Group were designed to have mice with similar weight in each group. To induce hepatocyte gene deletion, half of each litters (Rspo3 fl/fl mice) received AAV8-TBG-Null and the other half received a similar dose of AAV8-TBG-CRE. To induce Rspo3 gene hyperexpression, half of each litters (LratCre iDTRhet) received AAV8-CMV-GFP and the other half received a similar dose of AAV8-CMV-Rspo3.
Blinding	For mouse treatment and euthanasia as well as post euthanasia analysis such as (i) quantification by IHC and (ii) determination of gene expression by qPCR, the investigators were blinded. Investigators were not blinded for the analysis of single cell or single nucleus RNA-seq analyses. For immunoblotting, the investigators were not blinding when loading the gel to display the results in a logical way.

Reporting for specific materials, systems and methods

We require information from authors about some types of materials, experimental systems and methods used in many studies. Here, indicate whether each material, system or method listed is relevant to your study. If you are not sure if a list item applies to your research, read the appropriate section before selecting a response.

Materials & experimental systems

n/a	Involved in the study	
☐	☒	Antibodies
☐	☒	Eukaryotic cell lines
☒	☐	Palaeontology and archaeology
☐	☒	Animals and other organisms
☒	☐	Clinical data
☒	☐	Dual use research of concern
☒	☐	Plants

Methods

n/a	Involved in the study	
☒	☐	ChIP-seq
☐	☒	Flow cytometry
☒	☐	MRI-based neuroimaging

Antibodies

Antibodies used

Description of all antibodies used in the study are provided in the Materials&Methods and listed below:

In vitro studies:
RSPO3 neutralizing antibody (ProteoGenix, PX-TA1446)
Isotype control antibody (ProteoGenix, PTX17885)

Immunostaining studies:
Anti-Ki67 antibody (Abcam, ab16667)
Anti-Cyclin D1 (Abcam, ab134175)
Anti-CYP1A2 (Santa Cruz, sc-53241)
Anti-CYP2E1 (Abcam, ab28146)
Anti-RGN (Thermofisher, PA5-56057)
Anti-HAL (Sigma, HPA038547)
Anti-OAT (antibodies.com, A15120)
Anti-GS (Abcam, ab176562)

Multiplex IHC
Anti-CYP1A2 (Santa Cruz, sc-532410)
Anti-RGN (Thermofisher, PA5-56057)
Anti-GS (Abcam, ab176562)
Anti-CYP2F2 (Santa Cruz, sc-374540)
Anti-HNF4α (Thermofisher, MAI-199)
Anti-E-cadherin (Cell Signaling, 3195)
Anti-Na:K ATPase (Abcam, ab7671)
Anti-Rabbit IgG Alexa750 (Thermofisher, A21039)
Anti-Mouse IgG Alexa 647 (Cell Signaling, 4410S)

Spatial metabolomics study:
Anti-GS (Abcam, ab176562)

Immunoblotting studies:
Anti-ALDH2 (Proteintech, 15310-1-AP)
Aanti-RSPO3 (Proteintech, 17193-1-AP)
Anti-GAPDH (Sigma, G9295)
Anti-b actin (Sigma, A3854)
HRP anti-rabbit (Santa Cruz, sc-2004)

Flow cytometry
anti-CD16/32 (Tonbo, 2.4G2)
anti-CD45 (BD and BioLegend, clone 30-F11)
anti-CD19 (Tonbo, clone 1D3)

anti-CD3e (Tonbo, clone 145-2C11)
anti-CD4 (BD, clone RM4-5)
anti-CD8a (Tonbo, clone 53-6.7)
anti-NK1.1 (BD, clone PK136)
anti-CD11b (BD, clone M1/70)
anti-CD11c (BD, clone HL3)
anti-F4/80 (Tonbo, clone BM8.1)
anti-Ly6C (BioLegend, clone HK1.4)
anti-Ly6G (BioLegend, clone 1A8)
anti-B220 (BD, RA3-6B2)
anti-CD44 (Biolegend, IM7)
anti-CD64 (Biolegend, X54-5/7.1)
anti-CD80 (Tonbo, 16-10A1)
anti-CD86 (BD, GL1)
anti-VSIG4 (eBioscience, NLA14)
anti-MHCII (Tonbo, clone M5/114.15.2).
anti-CD3e (BD, clone 145-2C11)
anti-TCRβ (BD, clone H57-597)
anti-FOXP3 (eBioscience, FJK-16s)
anti-Ki67 (Thermo, clone SolA15)
anti-granzyme-B (BioLegend, clone QA16A02)

Validation

All antibodies are commercially available and have been validated by supplier.
The validation information of primary antibodies used for imunostaining and immunoblotting found on supplier's webpage is as follows:

Anti Ki-67 antibody (Suitable for: Flow Cyt (Intra), IHC-P, WB, mIHC, ICC/IF; Knockout validated; Reacts with: Mouse, Rat, Human; https://www.abcam.com/products/primary-antibodies/ki67-antibody-sp6-ab16667.html)
Anti-Cyclin D1 (Suitable for: WB, IP, ICC/IF, IHC-P; Reacts with: Mouse, Rat, Human; https://www.abcam.com/products/primary-antibodies/cyclin-d1-antibody-epr2241-c-terminal-ab134175.html?productWallTab=ShowAll)
Anti-CYP1A2 (Suitable for: WB, IP, IF and IHC(P); Reacts with: mouse, rat and human; https://www.scbt.com/p/cyp1a2-antibody-d15)
Anti-CYP2E1 (Suitable for: WB, ICC/IF; Reacts with: Mouse, Rat, Rabbit, Human; https://www.abcam.com/products/primary-antibodies/cytochrome-p450-2e1-antibody-ab28146.html)
Anti-RGN (Suitable for: WB, IHC; Reacts with:Human, Mouse, Rat; https://www.thermofisher.com/antibody/product/RGN-Antibody-Polyclonal/PA5-56057)
Anti-HAL (Suitable for: IHC; Reacts with:Human, Mouse, Rat; https://www.sigmaaldrich.com/US/en/product/sigma/hpa038547?srsltid=AfmBOooa3rc-NNbpKdIt1MwLxU3JZH4lH9kbvhlnQrLx_VM6fJ3s6Mhi)
Anti-OAT (Suitable for: WB, IHC, IP; Reacts with:Human, Mouse, Rat; https://www.antibodies.com/ornithine-aminotransferase-antibody-a15120)
Anti-GS (Suitable for: mIHC, IHC-Fr, WB, IHC-P; Knockout validated; Reacts with: Mouse, Rat, Human; https://www.abcam.com/products/primary-antibodies/glutamine-synthetase-antibody-epr13022b-ab176562.html?productWallTab=ShowAll)
Anti-CYP2F2 (Suitable for: WB, IP, IF and ELISA; Reacts with: mouse and rat; https://www.scbt.com/p/cyp2f2-antibody-f-9
Anti-HNF4α (Suitable for: WB, IHC, IHC (P), ICC/IF, Flow, ELISA, IP, ChIP, FN; Reacts with : Human, Mouse, Rat; https://www.thermofisher.com/antibody/product/HNF4A-Antibody-clone-K9218-Monoclonal/MA1-199)
Anti-E-cadherin (Suitable for: WB, IHC, IHC (P), IF, Flow; Reacts with : Human, Mouse; https://www.cellsignal.com/products/primary-antibodies/e-cadherin-24e10-rabbit-mab/3195)
Anti-Na:K ATPase (Suitable for: ICC/IF, IHC-P, WB; Reacts with: Mouse, Rat, Rabbit, Human, Pig; https://www.abcam.com/products/primary-antibodies/alpha-1-sodium-potassium-atpase-antibody-4646-ab7671.html)
Anti-Rabbit IgG Alexa750 (Suitable for: WB, ICC/IF, Flow; Reacts with: Rabbit; https://www.thermofisher.com/antibody/product/Goat-anti-Rabbit-IgG-H-L-Cross-Adsorbed-Secondary-Antibody-Polyclonal/A-21039)
Anti-Mouse IgG Alexa 647 (Suitable for: IF, Flow; Reacts with: Mouse; https://www.cellsignal.com/products/secondary-antibodies/anti-mouse-igg-h-l-f-ab-2-fragment-alexa-fluor-647-conjugate/4410)
Anti-ALDH2 (Suitable for: WB, IP, IHC, IF, CoIP, ELISA; Reacts with: Human, Mouse, Rat; https://www.ptglab.com/products/ALDH2-Antibody-15310-1-AP.htm)
Anti-RSPO3 (Suitable for: WB, IHC, IF, IP, ELISA; Reacts with:Human, Mouse, Rat; https://www.ptglab.com/products/RSPO3-Antibody-17193-1-AP.htm)
Anti-GAPDH (Suitable for: WB; Reacts with: rabbit, canine, rat, hamster, monkey, mouse, turkey, bovine, mink, human, chicken; https://www.sigmaaldrich.com/US/en/product/sigma/g9295)
Anti-b actin (Suitable for: WB; Reacts with:sheep, carp, feline, chicken, rat, mouse, Hirudo medicinalis, rabbit, canine, pig, human, bovine, guinea pig; https://www.sigmaaldrich.com/US/en/product/sigma/a3854?srsltid=AfmBOophnMsldF-su_vD-JDiPSVtGISuPLTuS3csBxH9uJHLOsBOIpT2)
HRP-anti-rabbit (Suitable for: WB; Reacts with: Rabbit; https://www.scbt.com/p/goat-anti-rabbit-igg-hrp)
anti-CD16/32 antibody (Suitable for: FC, IF, IP; Reacts with: Mouse; https://www.fishersci.com/shop/products/pure-ms-cd16-cd32-2-4g2-1mg/501055032)
anti-CD45 (Suitable for: FC; Reacts with: Mouse; https://www.biolegend.com/fr-ch/products/brilliant-violet-510-anti-mouse-cd45-antibody-7995)
anti-CD19 (Suitable for: Flow Cytometry; Reacts with: Mouse; https://cytekbio.com/products/percp-cyanine5-5-anti-mouse-cd19-1d3?variant=40581196709924)
anti-CD3e (Suitable for: Flow Cytometry, IHC, IHC-P, IHC-F, Flow, FN; Reacts with: Human, Mouse; https://www.thermofisher.com/antibody/product/CD3e-Antibody-clone-145-2C11-Monoclonal/45-0031-82)
anti-CD4 (Suitable for: Flow cytometry; Reacts with: Mouse; https://www.bdbiosciences.com/en-be/products/reagents/flow-cytometry-reagents/research-reagents/single-color-antibodies-ruo/buv737-rat-anti-mouse-cd4.612844)
anti-CD8a (Suitable for: Flow cytometry ; Reacts with: Mouse; https://cytekbio.com/products/apc-anti-mouse-cd8a-53-6-7?variant=40581236555812)
anti-NK1.1 (Suitable for: Flow cytometry ; Reacts with: Mouse; https://www.bdbiosciences.com/en-us/products/reagents/flow-cytometry-reagents/research-reagents/single-color-antibodies-ruo/buv395-mouse-anti-mouse-nk-1-1.564144)

anti-CD11b (Suitable for: Flow cytometry ; Reacts with: Mouse, Human; https://www.bdbiosciences.com/en-us/products/reagents/flow-cytometry-reagents/research-reagents/single-color-antibodies-ruo/bv650-rat-anti-cd11b.563402)
anti-CD11c (Suitable for: Flow cytometry, Immunohistochemistry-frozen, Immunohistochemistry-formalin, Immunohistochemistry-paraffin, Immunohistochemistry-zinc-fixed; Reacts with: Mouse; https://www.bdbiosciences.com/en-us/products/reagents/flow-cytometry-reagents/research-reagents/single-color-antibodies-ruo/buv737-hamster-anti-mouse-cd11c.612796)
anti-F4/80 (Suitable for: Flow Cytometry; Reacts with: Mouse; https://cytekbio.com/products/apc-anti-mouse-f4-80-antigen-bm8-1?variant=40581236424740)
anti-Ly6C (Suitable for: FC, IHC-F; Reacts with: Mouse; https://www.biolegend.com/fr-lu/products/brilliant-violet-510-anti-mouse-ly-6c-antibody-8726)
anti-Ly6G (Suitable for:FC; Reacts with: Mouse; https://www.biolegend.com/nl-nl/products/purified-anti-mouse-ly-6g-antibody-4767?GroupID=BLG7232)
anti-B220 (Suitable for:Flow cytometry; Reacts with: Mouse, Human; https://www.bdbiosciences.com/en-us/products/reagents/flow-cytometry-reagents/research-reagents/single-color-antibodies-ruo/buv496-rat-anti-mouse-cd45r-b220.612950)
anti-CD44 (Suitable for:FC; Reacts with: Mouse, Human; https://www.biolegend.com/en-ie/products/brilliant-violet-650-anti-mouse-human-cd44-antibody-8923)
anti-CD64 (Suitable for:FC; Reacts with: Mouse; https://www.biolegend.com/fr-ch/products/pe-cyanine7-anti-mouse-cd64-fcgammari-antibody-10062)
anti-CD80 (Suitable for:Flow Cytometry; Reacts with: Mouse; https://cytekbio.com/products/fitc-anti-mouse-cd80-b7-1-16-10a1?variant=40581223252004)
anti-CD86 (Suitable for:Flow Cytometry; Reacts with: Mouse; https://www.fishersci.com/shop/products/anti-cd86-clone-gl1-bd-3/BDB563055)
anti-VSIG4 (Suitable for:WB, IHC-F, FC; Reacts with: Human, Mouse; https://www.thermofisher.com/antibody/product/VSIG4-Antibody-clone-NLA14-Monoclonal/17-5752-82)
anti-MHCII (Suitable for:Flow Cytometry; Reacts with: Mouse; https://cytekbio.com/products/violetfluor-450-anti-mouse-mhc-class-ii-i-a-i-e-m5-114-15-2?variant=40581180981284)
anti-CD3e (Suitable for:Flow Cytometry; Reacts with: Mouse; https://www.bdbiosciences.com/en-us/products/reagents/flow-cytometry-reagents/research-reagents/single-color-antibodies-ruo/buv496-hamster-anti-mouse-cd3e.612955)
anti-TCRβ (Suitable for:Flow Cytometry; Reacts with: Mouse; https://www.bdbiosciences.com/en-us/products/reagents/flow-cytometry-reagents/research-reagents/single-color-antibodies-ruo/bv711-hamster-anti-mouse-tcr-chain.563135)
anti-FOXP3 (Suitable for:IHC, IHC-F, ICC/IF, Flow; Reacts with: Bovine, Dog, Cat, Mouse, Pig, Rat; https://www.thermofisher.com/antibody/product/FOXP3-Antibody-clone-FJK-16s-Monoclonal/11-5773-82)
anti Ki-67 (Suitable for:IHC, IHC-P, IHC-PFA, IHC-F, ICC/IF, Flow, FN; Reacts with: Dog, Cynomolgus monkey, Human, Mouse, Non-human primate, Rat; https://www.thermofisher.com/antibody/product/Ki-67-Antibody-clone-SolA15-Monoclonal/56-5698-82)
anti-granzyme-B (Suitable for:ICFC; Reacts with: Human, Mouse; https://www.biolegend.com/nl-be/products/apc-anti-human-mouse-granzyme-b-recombinant-antibody-14429)

Eukaryotic cell lines

Policy information about cell lines and Sex and Gender in Research

Cell line source(s)	The mouse hepatocyte cell line (AML12) used in this study were obtained from ATCC. The human hepatic stellate cell line (LX-2) used in this study was obtained from Millipore.
Authentication	The cell lines were authenticated by the morphology.
Mycoplasma contamination	The cell lines were sporadically tested for mycoplasma contamination.
Commonly misidentified lines (See ICLAC register)	No commonly misindentified cell lines were used.

Animals and other research organisms

Policy information about studies involving animals; ARRIVE guidelines recommended for reporting animal research, and Sex and Gender in Research

Laboratory animals	All the mice were in a C57Bl/6 background with at least 5 backcrosses the exception of LratCre Wls floxed mice that were in a mixed background C57Bl/6 - 129/Sv, backcrossed twice to C57Bl/6..<br>The following strains were used for the experiment described in the manuscript:<br>LratCre, Lyve1Cre, Clec4fCre, Mx1Cre, Pdgfrβ-P2A-CreERT2, Cdh5-CreERT2, Tdtomato (TdTom) Ai14 reporter, Rosa26-HBEGF (iDTR), Rspo3 floxed, Wls floxed, Col1a1 floxed, Tgfbr1 floxed, Pdgfrb floxed, Hgf floxed and BALB/c mice.<br>The age for the mice are as below. LratCre/Tdtom/iDTR 7-34 weeks old, LratCre/Rspo3 floxed 7-42 weeks old (these include "aged mice"), Lyve1Cre/Rspo3 floxed 7-16 weeks old, Clec4fCre/Rspo3 floxed 8 weeks old, Mx1Cre/Col1a1 floxed 11-12 weeks old, LratCre/Wls floxed 8-11 weeks old, LratCre/Tgfbr1 floxed 9-10 weeks old, LratCre/Col1a1 floxed 9-11 weeks old, LratCre/Rspo3 floxed/HGF floxed 7-8 weeks old, Pdgfrβ-P2A-CreERT2/Rspo3 floxed 9-18 weeks old, Cdh5-CreERT2/Rspo3 floxed 11-16 weeks od.
Wild animals	No wild animals were used in the study.
Reporting on sex	All experiments were performed on male with the exception of:<br>-Some Rspo3 floxed mice were females as indicated in the figure legends.
Field-collected samples	No field collected samples were used in the study.
Ethics oversight	All animal procedures were performed with approval by Columbia University Institutional Animal Care and Use Committee

Ethics oversight

(protocolsAC-AABQ5565, AC-AABP3560 and AC-AABQ5566), the local institutional or the Vanderbilt University Institutional Animal Care and Use Committee (protocol M2000054-01) and in accordance with the Guide for the Care and Use of Laboratory Animals; or with approval from the governmental animal care and use committees Karlsruhe, Germany (in accordance with German national guidelines on animal welfare and the regulations of the regional council Karlsruhe under permit number G-251/20).

Note that full information on the approval of the study protocol must also be provided in the manuscript.

Plants

Seed stocks

N/A

Novel plant genotypes

N/A

Authentication

N/A

Flow Cytometry

Plots

Confirm that:

☒ The axis labels state the marker and fluorochrome used (e.g. CD4-FITC).

☒ The axis scales are clearly visible. Include numbers along axes only for bottom left plot of group (a 'group' is an analysis of identical markers).

☒ All plots are contour plots with outliers or pseudocolor plots.

☒ A numerical value for number of cells or percentage (with statistics) is provided.

Methodology

Sample preparation

Liver tissues were mechanically homogenised followed by an enzymatic digestion with 1 mg/ml of collagenase A (Roche, 10103578001) and 0.5 µg/ml DNase I (Roche, 10104159001) in isolation buffer (RPMI 1640, 5% FBS, 1% L-glutamine, 1% penicillin–streptomycin and 10 mM HEPES) for 45 min at 150 r.p.m. at 37°C. Cells were filtered through a 100-µm cell strainer, washed and separated in 2 parts to analyse the myeloid and the lymphocytes cell subsets. For the latter, cells were loaded onto a Percoll gradient (67% overlay with 40%) followed by red blood cell lysis using ammonium-chloride-potassium buffer and stained.

Instrument

Samples were analysed using a BD LSR Fortessa cell analyser.

Software

Flow cytometry analysis was performed using FlowJo (v.10.10.0).

Cell population abundance

For immune cells analysis, all the cells of the sample were sorted or analyzed.

Gating strategy

For immune cells analysis:
Debris exclusion by FSC-A/SSC-A. Dounlets were excluded using FSC-A/FSC-H, Life/Dead exclusion was performed using Ghost Dye Red 780 cell viavility reagent. Remaining cells were analyzed according to displayed markers and following the gating strategy provided in Supplementary information.

☒ Tick this box to confirm that a figure exemplifying the gating strategy is provided in the Supplementary Information.
