# Supplementary material for: Hepatic stellate cells control liver zonation, size and functions via R-spondin 3
Source: Nature. 2025 Mar 12;640(8059):752–61. doi: 10.1038/s41586-025-08677-w (PMC12003176; doi:10.1038/s41586-025-08677-w)
Supplement: Supplementary file 4 — Supplementary Tables 1–12. [file 41586_2025_8677_MOESM4_ESM.zip › 2024-02-04354B-s4/SupplementaryTablelegends.docx]

**SUPPLEMENTARY TABLES**

**Supplementary Table 1.** Bulk RNA-seq analysis of JEDI and iDTR HSC-depleted livers.

**Supplementary Table 2.** Top 100 genes downregulated in JEDI and iDTR HSC-depleted livers after removal of genes with > 3logFC enrichment in HSCs.

**Supplementary Table 3.** Top 15 CTNNB1-regulated genes (from mice with hepatocyte-specific CTNNB1 ko) in JEDI and iDTR HSC-depleted livers.

**Supplementary Table 4.** Ligand-receptor interactions HSC to hepatocyte in mouse liver snRNA-seq ranked by interaction score.

**Supplementary Table 5.** Bulk RNA-seq analysis comparing Rspo3-floxed livers to livers with constitutive Rspo3 knockout in HSCs by LratCre (Rspo3ΔHSC).

**Supplementary Table 6.** Bulk RNA-seq analysis compared Rspo3-floxed livers to livers with constitutive Rspo3 knockout in endothelial cells by Lyve1-Cre (Rspo3ΔEC).

**Supplementary Table 7.** Bulk RNA-seq analysis comparing Rspo3-floxed livers to livers with constitutive Rspo3 knockout in HSCs by LratCre (Rspo3ΔHSC) from 32-34 weeks old aged mice.

**Supplementary Table 8.** In silico metabolomic analysis in HSC-depleted Rspo3ΔHSC and CTNNB1ΔHep livers.

**Supplementary Table 9.** Biocrates 500XL metabolomics data from livers of Rspo3fl/fl and Rspo3ΔHSC mice (log-transformed).

**Supplementary Table 10.** Human snRNA-seq samples.

**Supplementary Table 11.** Ligand-receptor interactions HSC to hepatocyte in human liver snRNA-seq ranked by interaction score.
